# Males’ Awareness of Female and Male Contraception Methods, Information, Outreach, and Acquisition Locations in Abidjan, Côte d’Ivoire, Nairobi, Kenya, and Lagos, Nigeria

**DOI:** 10.1016/j.jadohealth.2022.03.013

**Published:** 2022-09

**Authors:** Arik V. Marcell, Meagan E. Byrne, Nathalie Yao-N'dry, Mary Thiongo, Peter Gichangi, Funmilola M. OlaOlorun, Scott Radloff, Philip A. Anglewicz, Amy O. Tsui

**Affiliations:** a Division of Adolescent/Young Adult Medicine, Department of Pediatrics, Johns Hopkins School of Medicine, Baltimore, Maryland; b Department of Population, Family and Reproductive Health, Johns Hopkins Bloomberg School of Public Health, Baltimore, Maryland; c Department of Population, Family and Reproductive Health, Bill & Melinda Gates Institute for Population and Reproductive Health, Johns Hopkins Bloomberg School of Public Health, Baltimore, Maryland; d Association Ivoirienne pour le Bien-Etre Familial (AIBEF), Abidjan, Côte d’Ivoire; e International Centre for Reproductive Health, Mombasa, Kenya; f Technical University of Mombasa, Mombasa, Kenya; g Department of Public Health and Primary Care, Faculty of Medicine and Health Sciences, Ghent University, Ghent, Belgium; h Department of Community Medicine, College of Medicine, University of Ibadan, Ibadan, Oyo State, Nigeria

**Keywords:** Young men, Male adolescent, Young adult male, Contraception method awareness, Contraception information source, Location to obtain contraception

## Abstract

**Purpose:**

The aim of this study is to describe modern female and male method awareness, information sources, outreach exposures, and acquisition source awareness among young men aged 15–24 by sexual behavior status in sub-Saharan Africa.

**Methods:**

Cross-sectional surveys were conducted with unmarried, young men aged 15–24 recruited via respondent-driven sampling in Abidjan, Côte d’Ivoire (n = 1,028), Nairobi, Kenya (n = 691), and Lagos, Nigeria (n = 706). Descriptive statistics characterized contraception awareness of male and female methods and information sources, outreach exposures, acquisition source awareness, and preferred contraception source. Multivariate regressions characterized factors associated with awareness of each method.

**Results:**

Majority of respondents were aged 15–20 (59%), sexually active (65%), and had secondary or more education (89%). Awareness was low for all methods (short-acting reversible contraception, 47%; emergency contraception, 35%; long-acting reversible contraception, 32%; withdrawal, 18%), except condoms (85%). Respondents reported low levels of contraception information sources, recent outreach exposures, and acquisition location awareness that varied by sexual behavior (higher among sexually active than nonsexually active respondents). Multivariate analyses demonstrated common factors associated across awareness of all methods included information sources (teacher, friend, Internet, social media for all respondents; pharmacist for sexually active respondents) and acquisition locations (private healthcare, pharmacy, market/store for all respondents; public healthcare, mobile clinic, faith-based organizations for sexually active respondents). Sexually active respondents’ rank order for preferred contraception source was doctors/nurses followed by teachers, friends, mothers, and fathers; and for nonsexually active respondents’ rank order was teachers followed by friends, mothers, doctors/nurses, and health centers.

**Discussion:**

Findings have implications for increasing young men’s method awareness, specific sources, and settings to target contraceptive outreach.

**Implications and Contribution**

Little is known about young men’s modern hormonal contraception awareness, information sources, and acquisition points in Sub-Saharan Africa. Findings demonstrate low levels of method awareness, except for condoms, lack of awareness about contraception information sources, exposure to recent outreach, and acquisition locations, and common associated factors with awareness of all methods, including information sources and acquisition locations.

Despite a worldwide decline in unintended pregnancies, in 2017 almost half of all pregnancies in developing regions were estimated to be unintended, and approximately 214 million reproductive-aged women who wanted to avoid pregnancy were not using a modern hormonal method [1,2]. In sub-Saharan Africa (SSA), rates of modern method use were low, especially among women aged 15–24 [3,4].

The Guttmacher-Lancet Commission’s integrated definition of sexual and reproductive health (SRH) and rights recommends that SRH information and services, especially related to contraception, be accessible and affordable to all individuals who need them [2]. It is critical to engage young men as partners in family planning (FP) and SRH since they often may not have the needed information and services [2]. Research and programs in this area have focused on women’s modern hormonal method access with less attention to research and programming for young men [5]. Gaining a better understanding of young men’s modern female hormonal method awareness, in addition to male methods, information sources, and acquisition points, will be important to engaging them as partners in FP.

Past research in SSA has reported on young people’s awareness about contraceptive methods, but not specifically for young men or on young men’s awareness about modern female hormonal methods, such as newer long-acting reversible contraception (LARC) inclusive of implants and intrauterine devices [[6], [7], [8]]. Overall, this work, conducted 10 years ago or more, demonstrated that a high proportion of young people were aware of condoms (ranging from 42% to 91%), but lower proportions were aware of other methods, such as pills (24%–31%), injectables (3%–26%), withdrawal (4%), and emergency contraception (EC) (6%) [[6], [7], [8]]. This past work also did not examine the degree to which young men’s method awareness differed by their sexual behavior status. Understanding awareness of modern hormonal method among young men who are sexually active as well as those who have not yet initiated sex but may be on the brink of engaging in sexual activity will be important for developing developmentally appropriate education and outreach strategies and reducing levels of nonuse of contraception at first sex.

A number of studies have examined young people’s contraception information sources [[5], [6], [7],^9^,^10^], yet none have described findings for young men. These studies’ findings are also inconsistent since information sources were assessed differently across studies among varying age ranges of youth, and some studies are limited in their use of double-barreled measures. Television/radio and friends were identified as “contraception information sources” by 15- to 20-year-olds in Ghana [6]. Radio followed by newspaper and television were identified as the “most popular sources of FP information” by 15- to 19-year-olds in Tanzania [10]. Parents, peers, and teachers were identified by 15- to 19-year-olds in Ethiopia as individuals with whom they “discussed reproductive health” [9]. Mass media, teachers, and friends were identified as “information sources used for contraception, sexually transmitted infections, or human immunodeficiency virus” by 12- to 14-year-olds in Burkina Faso, Ghana, Malawi, and Uganda [7]. These studies also did not describe young men’s awareness of locations to acquire contraception.

Performance Monitoring and Accountability (PMA) Agile, a program within the broader PMA Project, collected data on contraception method awareness and access and reproductive behaviors among urban unmarried males and females aged 15–24 living in Abidjan, Côte d’Ivoire, Nairobi, Kenya, and Lagos, Nigeria. Similar to many countries in SSA, these settings have burgeoning youth populations, with over half living in urban cities [11]. In Abidjan, Côte d’Ivoire, age at first sex among urban women occurred almost 4 years on average before age of first contraceptive use [12,13], and only 30% of all females aged 15–19 years reported current contraception use, primarily EC, and male condoms [12]. In Lagos, Nigeria, only 28% and 37% of unmarried women aged 15–19 and 20–24 reported using any current contraceptive method, respectively; the modern contraception prevalence rate among married women in Lagos is 29% [14]. Although in Nairobi, Kenya modern contraception prevalence rate is higher—estimated at 58%—SRH issues for youth persist, including adolescent pregnancy [15]; only 13% of females under 18 reported ever using contraception [16].

Exploring factors associated with young men’s awareness of female as well as male methods, such as specific contraception information sources, outreach exposures, and acquisition sources, controlling for age, educational level, and city, will be important to inform common targets for contraception education and outreach strategies with young men to support contraception use. Exploring such associations stratified by young men’s sexual behavior status will also assist in identifying specific outreach approaches to engage young men who are not yet sexually active as well as for those who are.

Addressing gaps in the literature, this study’s main goal was to describe awareness about female modern methods, in addition to male methods, and contraception information sources, outreach exposures, and acquisition source awareness among young men aged 15–24 by sexual behavior status from three cities in SSA: Abidjan, Côte d’Ivoire, Nairobi, Kenya, and Lagos, Nigeria. This study’s secondary goal was to explore associations between young men’s awareness of each method with each information source, outreach exposure, and acquisition source awareness by sexual behavior status unadjusted and adjusted for participants’ age group, highest grade in school completed, and city location.

## Methods

### Study procedures

Data for this secondary data analysis come from cross-sectional surveys conducted by PMA in Abidjan, Côte d’Ivoire (August 10, 2018 to November 10, 2018), Nairobi, Kenya (June 21, 2019 to August 14, 2019), and Lagos, Nigeria (February 28, 2020 to March 21, 2020) with unmarried individuals aged 15–24 which covered method awareness, access, and reproductive behaviors. Selection of the three study cities was based on unmet need related to adolescent pregnancy prevention, research capacity, and geographic diversity. Participants with at least 1 year of local residence (i.e., Abidjan region, Nairobi county, and Lagos state) were recruited via respondent-driven sampling (RDS). Use of RDS that uses a peer-to-peer recruitment approach was chosen to recruit unmarried young people given feasibility concerns of clinic-based sampling and confidentiality concerns about household-based sampling on hidden or stigmatized topics, such as sexual activity and contraceptive use. Eligible seeds were purposefully selected on demographic characteristics, including sex, age, educational status, and neighborhood of residence [17]. Seeds catalyzed peer-to-peer recruitment via coupons (up to three per person) until the target sample size was achieved. Following determination of eligibility and informed consent (parental consent for minors under 18 was waived in all studies) using a Coupon Manager form administered by study staff, seed participants, and subsequent recruits completed a self-administered survey using a handheld tablet to maximize confidentiality and accuracy, and minimize bias [18]. Staff assisted participants in cases of limited literacy or tablet unfamiliarity. Study procedures were conducted in the respondents’ language of choice: French in Abidjan, Swahili or English in Nairobi, and Yoruba or English in Lagos. Additional information about this study’s RDS recruitment strategy is described elsewhere [3,4,19]. Procedures were approved by Institutional Review Boards at Johns Hopkins Bloomberg School of Public Health and local review boards (Comité National d’Ethique des Sciences de la Vie et de la Santé of the Ministry of Health and Public Hygiene, Côte d’Ivoire; the Ethics Review Committee at Kenyatta National Hospital/University of Nairobi, Kenya; and the Health Research and Ethics Committee at the Lagos State University Teaching Hospital). The current analysis is restricted to the young male sample (Total, n = 2,425; Abidjan, n = 1,028; Nairobi, n = 691; and Lagos, n = 706).

### Measures

#### Sexual behavior status

Respondents were asked whether they had ever gotten a girl pregnant and if answered no, whether they had ever had sexual intercourse (defined as vaginal penetrative sex). Based on an affirmative response to either question, the preprogrammed survey generated a combined measure for sexual activity coded as sexually active or not.

#### Contraception method awareness

Respondents were asked to mark what methods of contraception they had ever heard of: male condom, withdrawal, implant, intrauterine device, injectables, pill, and EC (each coded as yes/no; responses for implant and intrauterine device were combined as LARC methods and injectable and pill as short-acting reversible contraception [SARC] methods, respectively).

#### Contraception information source

Respondents were asked to mark the following sources from whom they learned about contraception: mother, father, other relative(s), brother(s), sister(s), friend(s), doctor/nurse, pharmacist/shop keeper, health worker, teacher, religious leader, Internet/web, social media, other source, or no one (each coded as yes/no). Respondents were also asked to select where or from whom they would prefer to have received information on this topic.

#### Recent FP outreach exposure

Respondents were asked whether they attended a community event where FP was favorably discussed in the last year (coded as yes/no for “FP community exposure”); heard the following authorities speak publicly in favor of FP in the last year: religious leaders, civic/community leaders, state/municipal leaders, and governmental officials at the national level (each coded as yes/no for “FP authority exposure”); and had FP media exposure in past months: radio, TV, newspaper/magazine, brochure/leaflet/flyer, voice/short message service on mobile phone, poster/billboard, or social media site (each coded as yes/no for “FP media exposure”).

#### Contraception acquisition source awareness

Respondents were asked to mark awareness of the following sources where they can obtain contraception: private healthcare setting (university, private hospital), public healthcare setting (urban, government health center), FP clinic, pharmacy, market/store, nonprofit organization, fieldworker (e.g., community health outreach worker or volunteer), mobile clinic, faith-based organization/church, friend/relative, or other place (each coded as yes/no).

#### Background characteristics

Respondents’ age was coded by age group as 15–17, 18–20, or 21–24. Highest grade in school completed was coded as being in primary school or less, secondary school, or more than secondary school. City location was coded as Abidjan, Côte d’Ivoire; Nairobi, Kenya; and Lagos, Nigeria.

### Data analysis

All reported frequencies and analyses are weighted according to established procedures and incorporated the complex survey design including accounting for clustering by node (participants recruited by the same recruiter). The RDS estimates were weighted using RDS-II (Volz-Heckathorn) weights, developed using RDS-Analyst software [20], accounting for differences in reported network size as proxy for likelihood of receiving a coupon. A postestimation weight was used in addition to the RDS-II weight based on the latest national Demographic and Health Survey data to account for modest demographic differences between the RDS sample and underlying population of unmarried 15- to 24-year-olds in each site [[13], [14], [15]]. Data management was conducted using SPSS 25 (Armonk, NY) and analyses were conducted in Stata 16 (College Station, TX).

We began analyses with frequencies for the overall sample and then cross-tabulations by sexual behavior status for method awareness, outreach exposure, acquisition source awareness, preferred contraception source, and background characteristics (Tables 1 and 2; and Table A1 for method awareness by city and sexual behavior status). Next, we conducted bivariate associations using weighted log binomial regression models to examine how each of these variables varied by young men’s sexual behavior status (Table 1). We then examined whether the rank order of preferred contraception source varied by sexual behavior status using the Mann-Whitney *U*-test (Table 2). Finally, associations were assessed separately for awareness of each method with each contraception source, outreach exposure, and acquisition source awareness unadjusted and adjusted for participants’ background characteristic using weighted log binomial regression models (Table 3 summarizes all findings; Tables A2–A5 provides detailed findings). *p*-Values were considered statistically significant at *p* < .05.

**Table 1:** Young men’s contraception method awareness, information source, recent exposure, awareness of locations to obtain, and background characteristics for total sample and by sexual behavior status

	Total sample		Sexually active		Never sexually active^a^	
	n^b^	%^c^	n^b^	%^c^	n^b^	%^c^
Contraception method awareness						
Male method						
Condom	2,084	84.7	1,384	87.4	700	79.7∗∗
Withdrawal	488	17.7	370	20.3	118	12.7∗∗
Female method						
Long-acting reversible contraception	749	32.1	515	34.0	234	28.6
Short-acting reversible contraception	1,152	46.7	785	50.7	367	39.2∗∗
Emergency contraception	798	35.1	611	42.5	187	21.3∗∗∗
Contraception information source						
Mother	679	22.4	451	22.5	228	22.1
Father	456	14.7	315	15.2	141	13.8
Other relative(s)	316	10.6	225	11.1	91	9.7
Brother(s)	496	17.6	365	17.6	131	17.5
Sister(s)	333	11.4	244	12.5	89	9.4
Friend(s)	1,093	45.4	760	49.9	333	37.0∗∗∗
Doctor/nurse	586	20.8	425	21.1	161	20.2
Pharmacist/shopkeeper	243	7.6	185	9.4	58	4.3∗∗
Health worker	496	16.2	368	18.9	128	11.1∗∗
Teacher	1,114	37.2	732	33.9	382	43.4∗∗
Religious leader	179	5.4	121	5.5	58	5.3
Internet/web	515	17.2	388	21.2	127	9.7∗∗∗
Social media (Facebook, WhatsApp)	520	19.2	381	23.1	139	11.9∗∗∗
Other source	62	2.0	42	1.8	20	2.5
None	19	0.6	10	0.4	9	1.0
Recent FP outreach exposure						
FP community exposure, last year	883	36.0	602	38.2	281	32.0
FP authority exposure, last year						
Religious leader	550	22.9	346	21.8	204	25.0
Civic/community leaders	496	21.4	337	22.2	159	20.0
State or municipal leaders	164	4.7	96	3.8	68	6.5
Governmental official (National level)	720	30.0	486	31.7	234	26.8
FP media exposure, past months						
Radio	1,370	62.7	922	66.6	448	55.4∗∗
Television	1,692	68.3	1,122	71.0	570	63.1∗
Newspaper/magazine	1,220	49.2	801	52.6	419	43.0∗
Brochure, leaflet, flyer	752	28.0	509	30.2	243	23.9
Voice or SMS on mobile phone	512	21.3	320	20.4	192	22.9
Poster/billboard	1,366	57.0	921	62.7	445	46.2∗∗∗
Social media site	1,600	68.3	1,068	74.8	532	56.1∗∗∗
Contraception acquisition source awareness						
Private healthcare setting	658	26.0	487	30.3	171	17.9∗∗∗
Public healthcare setting	985	45.3	738	52.0	247	32.8∗∗∗
Family planning clinic	629	24.0	453	25.0	176	22.1
Pharmacy	1,103	43.7	828	50.8	275	30.3∗∗∗
Market/store	473	16.3	362	19.5	111	10.3∗∗∗
Nonprofit organization	124	3.8	92	4.8	32	1.8∗∗
Fieldworker/attendant community	168	8.0	129	8.9	39	6.4
Mobile clinic	157	6.7	123	7.9	34	4.6
Faith-based organization/church	45	1.5	39	1.3	6	1.9
Friend/relative	305	9.8	244	11.6	61	6.4
Other location	50	1.3	38	1.5	12	1.1
Background characteristics						
Age group						
15–17 (Reference)	716	25.7	297	16.6	419	42.7
18–20	961	32.7	660	32.6	301	32.7∗∗∗
21–24	748	41.7	603	50.7	145	24.6∗∗∗
Sexual behavior status						
Not sexually active	865	34.7	–	–	865	100.0
Sexually active	1,560	65.3	1,560	100.0	–	–
Highest grade in school completed^d^						
Primary school or less (Reference)	88	10.8	48	10.0	40	12.2
Secondary	1,499	66.9	850	62.5	649	75.0
More than secondary	823	21.7	654	27.0	169	11.8∗∗
City location						
Abidjan, Côte d’Ivoire (Reference)	1,028	27.1	738	26.9	290	27.6
Nairobi, Kenya	691	49.9	479	54.3	212	41.6
Lagos, Nigeria	706	23.0	343	18.8	363	30.9∗∗

FP = family planning; SMS = short message service.

∗*p* < .05; ∗∗*p* < .01; ∗∗∗*p* < .001.

a Weighted log binomial regression models examined bivariate associations of each contraception method awareness, contraception source/location to obtain, recent contraception outreach exposure, and background characteristics with young men’s sexual behavior status.

b Unweighted.

c Weighted.

d Missing: n = 15 cases.

**Table 2:** Preferred contraception source by sexual behavior status^a^

Information source	Sexually active			Never sexually active		
	n^b^	%^c^	Rank	n^b^	%^c^	Rank
Doctor/nurse	165	13.0	1	93	11.4	4
Teacher	323	12.9	2	170	18.9	1
Friend	186	12.8	3	107	14.1	2
Mother	217	11.2	4	143	12.0	3
Father	114	9.9	5	52	6.6	6
Youth	73	5.5	6	46	5.0	7
Health center	48	5.2	7	34	7.9	5
Social media	43	4.5	8	8	1.1	14
Brother	71	3.4	9	34	4.6	8
Partner	31	3.4	9	14	1.4	13
Community health worker	64	3.2	10	27	2.3	11
Internet	54	3.0	11	17	1.6	12
None	40	2.1	12	34	3.8	9
Health fair	17	1.6	13	4	0.4	20
TV/Radio/Film	14	1.4	14	7	0.6	18
Other relatives	16	1.3	15	7	0.7	17
Don’t know	10	1.1	16	5	0.7	17
Sister	24	0.9	17	27	2.6	10
Billboard	13	0.9	17	4	0.7	17
Religious leader	11	0.9	17	11	1.0	15
Pharmacist	9	0.8	18	4	0.1	21
SMS	6	0.5	19	4	0.5	19
After school program	6	0.4	20	7	0.8	16
Book	5	0.2	21	6	1.1	14

SMS = short message service; TV = television.

a Mann-Whitney test found that young men’s rank order of preferred contraception source varied by sexual behavior status (*p* = .031).

b Unweighted.

c Weighted.

**Table 3:** Summary of factors associated with young men’s awareness of male and female contraception methods and emergency contraception by sexual behavior status^a^

	Male methods				Female methods				Emergency contraception		Summary pattern
	Condoms		Withdrawal		LARC		SARC				
	SA	NSA	SA	NSA	SA	NSA	SA	NSA	SA	NSA	
Contraception information source											
Mother											
Father					+						
Other relative(s)			+		+		+		+		Mainly SA
Brother(s)							+				
Sister(s)										+	
Friend(s)	+	+	+	+	+		+	+	+	+	All methods
Doctor/nurse	+	+			+		+		+		SA and female methods
Pharmacist/shop keeper	+		+	+		+	+		+		Mainly SA
Health worker				+	+	+	+	+			Mainly female methods
Teacher	+	+	+	+	+	+	+	+	+	+	All methods
Religious leader		+			+						
Internet/web	+	+	+	+	+	+	+	+	+	+	All methods
Social media (Facebook, WhatsApp)	+	+	+	+	+		+	+	+	+	All methods
Recent FP outreach exposure											
FP community exposure, last year			−							+	
FP authority exposure, last year											
Religious leader											
Civic/community leaders					+		+				SA and female methods
State or municipal leaders											
Governmental official (National level)											
FP media exposure, past months											
Radio											
Television	+		+		+						SA
Newspaper/magazine					+			+			
Brochure, leaflet, flyer					+						
Voice or SMS on mobile phone											
Poster/billboard				+	+			+	+		
Social media site							+				
Contraception acquisition source awareness											
Private healthcare setting	+	+	N/A	N/A	+	+	+	+	+	+	All methods
Public healthcare setting	+	+	N/A	N/A	+		+		+		Mainly SA
Family planning clinic		+	N/A	N/A	+	+	+	+	+	+	Mainly female methods
Pharmacy	+	+	N/A	N/A	+	+	+	+	+	+	All methods
Market/store	+	+	N/A	N/A	+	+	+	+	+	+	All methods
Nonprofit organization			N/A	N/A	+	+	+	+	+		Mainly female methods
Fieldworker		+	N/A	N/A	+	+	+	+	+	+	Mainly female methods
Mobile clinic	+		N/A	N/A	+		+		+		SA
Faith-based organization/church	+		N/A	N/A	+		+		+		SA
Friend/relative	+	+	N/A	N/A	+	+	+	+	+	+	All methods

+ = positive relationship in adjusted log binomial regression models; − = negative relationship in adjusted log binomial regression models; FP = family planning; LARC = long-acting reversible contraception; N/A = not applicable; NSA = never sexually active; SA = sexually active; SARC = short-acting reversible contraception; SMS = short message service.

a See Appendix for results of weighted log binomial regression models examining associations of each contraception information source, recent FP outreach exposure, contraception location acquisition awareness with each modern method awareness, respectively, unadjusted and adjusted for participants’ background characteristics.

## Results

The analytic sample’s age composition included 26% of 15- to 17-year-olds, 33% of 18- to 20-year-olds, and 42% of 21- to 24-year-olds (Table 1). The majority of young men were sexually active (65%) and had completed secondary (66%) or more education (22%).

The majority of young men reported condom awareness (85%); 47% were aware of SARCs, 35% of EC, 32% of LARCs, and 18% of withdrawal (Table 1). Young men’s method awareness varied by sexual behavior status for all methods, except LARCs (all *p* < .01), with significantly higher proportions of sexually active than never sexually active young men being aware of each method.

Young men’s most frequent information sources included friends (45%), teachers (37%), mothers (22%), and doctors/nurses (21%). Some sources varied by sexual behavior status; significantly higher proportions of sexually active young men reported friends, pharmacists, health workers, Internet, and social media (all *p* < .01), whereas significantly higher proportions of never sexually active young men reported teachers (*p* < .01).

About one third (36%) of young men attended community events in last year where FP was favorably discussed. One fifth heard the following people speak publicly in favor of FP: government officials (30%), religious leaders (23%), and civic/community leaders (21%). Almost half or more reported past month FP exposure on the TV (68%), social media site (68%), radio (63%), poster/billboard (57%), and newspaper/magazine (49%). Some young men’s FP media exposures varied by sexual behavior status; higher proportions of sexually active young men reported radio, TV, newspaper/magazine, poster/billboard, and social media sources and higher proportions of never sexually active young men reported state/municipal leaders favorably discussing FP (all *p* < .05).

Less than half of young men were aware of the following contraception acquisition sources: public healthcare (45%), pharmacy (44%), private healthcare (26%), and FP clinic (24%) settings, with awareness varying by young men’s sexual behavior status. Higher proportions of sexually active young men were aware that they could obtain contraception from private and public healthcare, pharmacy, market/store, and nonprofit organization settings, and friends/relatives (all *p* < .05).

Young men’s preferred contraception sources significantly varied by sexual behavior status (*p* = .031) (Table 2). Rank order for sexually active young men included first doctors/nurses followed by teachers, friends, mothers, and fathers, respectively, and rank order for never sexually active young men included first teachers followed by friends, mothers, doctors/nurses, and health centers.

### Common factors associated with young men’s contraception method awareness

#### Condoms

Regardless of sexual behavior status, greater condom awareness was associated with friend, doctor/nurse, teacher, Internet, and social media information sources; and awareness of private and public healthcare, pharmacy, market/store, and friend/relative acquisition sources (all *p* < .05) (Table 3 and Tables A2–A4). Greater condom awareness among sexually active young men was associated with pharmacy as an information source; television media exposure; and awareness of mobile clinic and faith-based organization/church acquisition sources; and among never sexually active young men was associated with religious leader as an information source and awareness of fieldworker as an acquisition source (all *p* < .05).

#### Withdrawal

Regardless of sexual behavior status, greater withdrawal awareness was associated with friend, pharmacy, teacher, Internet, and social media information sources (all *p* < .05) (Table 3 and Tables A2 and A3). Greater withdrawal awareness among sexually active young men was associated with “other relative” as an information source; and television media exposure; and among never sexually active young men was associated with health worker as an information source (all *p* < .05). Lower withdrawal awareness among sexually active young men was associated with favorable community discussion about FP (*p* < .05).

#### Long-acting reversible contraception

Regardless of sexual behavior status, greater LARC awareness was associated with pharmacy, health worker, teacher, and Internet information sources; and awareness of private healthcare and FP clinic, pharmacy, market/store, nonprofit organization, fieldworker, and friend/relative acquisition sources (all *p* < .05) (Table 3 and Tables A2–A4). Greater LARC awareness among sexually active young men was associated with father, other relative, friend, doctor/nurse, religious leader, and social media information sources; civic/community leaders speaking publicly in favor of FP; TV, magazine, brochure, and poster media exposures; and awareness of public healthcare, mobile clinic, and faith-based organization/church acquisition sources (all *p* < .05).

#### Short-acting reversible contraception

Regardless of sexual behavior status, greater SARC awareness was associated with friend, teacher, Internet, and social media information sources; and awareness of private healthcare, FP clinic, pharmacy, market/store, nonprofit organization, fieldworker, and friend/relative acquisition sources (all *p* < .05) (Table 3 and Tables A2–A4). Greater SARC awareness among sexually active young men was associated with other relative, brother, doctor/nurse, pharmacy, and health worker information sources; civic/community leaders speaking publicly in favor of FP; social media exposure; and awareness of public healthcare, mobile clinic, and faith-based organization/church acquisition sources; and among never sexually active young men was associated with religious leader as an information source and magazine and poster media exposures (all *p* < .05).

#### Emergency contraception

Regardless of sexual behavior status, greater EC awareness was associated with friend, teacher, Internet, and social media information sources; and awareness of private healthcare, FP clinic, pharmacy, market/store, fieldworker, and friend/relative acquisition sources (all *p* < .05) (Table 3 and Tables A2–A4). Greater EC awareness among sexually active young men was associated with other relative, doctor/nurse, and pharmacy information sources; and awareness of public healthcare, nonprofit organization, mobile clinic, and religious leader acquisition sources; and among never sexually active young men was associated with sister as an information source and favorable community discussion about FP (all *p* < .05).

#### Background characteristics

Greater method awareness was also consistently associated with young men’s age group and city location (Table A5). Among sexually active young men, greater condom, LARC, and EC awareness was associated with older than younger age groups and regardless of sexual behavior status, lower awareness across all methods was observed in Lagos, Nigeria.

## Discussion

This study found that young men recruited from three cities in SSA using RDS approach had generally low male and female modern hormonal method awareness, including EC. Relatively low proportions of young men reported having contraception information sources, recent outreach exposures, and acquisition source awareness. Higher proportions of sexually active than never sexually active young men were aware of female and male methods, contraception information sources, outreach exposures, and acquisition sources. Preferred contraception information source rankings also varied by young men’s sexual behavior status. Findings examining factors associated with young men’s type-specific contraception method awareness highlighted common contraception information and acquisition sources for program planners working with young men to increase contraceptive outreach, particularly through teachers, friends, social media, and pharmacists; and to increase supply, particularly through private and public sector, pharmacy, mobile clinic, market/store, and faith-based organization settings. Findings highlight the need for strategies to improve young men’s awareness about all modern male and female methods.

This is one of the first studies situated in SSA to describe young men’s modern hormonal method awareness and extends prior work with young people that found high condom awareness, but lower awareness of other methods [[6], [7], [8]]. This study extends past work in that it specifically examined young men’s method awareness by also their sexual behavior status. Current findings demonstrate substantial method awareness gaps that need to be addressed to more effectively engage young men as partners in FP, although greater contraception awareness and knowledge may be necessary but not sufficient to improve contraceptive practices [21]. Interventions in SSA teaching young people about female methods, including EC, will need to expand beyond focusing just on increasing adult men’s condom use and adult couple’s contraceptive use [22,23]. Past work demonstrates that contraception use is improved when partners are communicating about this together [23] and prior analyses from these data describe few young men believe preventing pregnancy is only their partner’s responsibility [3,4,19]. Hardee et al. [24] outlined 10 key considerations in programming for men as FP users in SSA, including providing information and services to men and boys where and when they need it and teaching adolescent boys about pregnancy prevention and healthy sexual relationships. Access to this type of comprehensive sex education (CSE) is also important for supporting adolescent boys’ socioemotional development and addressing gender and power issues that can lead to better health outcomes [[25], [26], [27]]. Prior analyses from these data also demonstrate that city-specific contextual factors may have contributed to overall study findings [3,4,19]. For example, adolescents in Lagos, Nigeria reported higher rates of stigma-based reasons for relying on someone else to get contraception than adolescents from other settings. Future work should further examine city-specific factors that can explain current study findings.

Findings about young men’s information sources are generally consistent with prior work that has not reported findings stratified by sex [[5], [6], [7],^9^] and extends this work by examining a range of contraception information sources, media exposures, and acquisition sources. Less than half of young men in the current study endorsed any information source or outreach exposure, except for some FP media exposures (i.e., radio, television, billboards, social media). Few young men also reported contraception acquisition source awareness, except for about half of sexually active young men who were aware of public healthcare and pharmacy settings. Strategies are needed to improve young men’s overall access to accurate and reliable contraception information and acquisition sources.

This study found that young men’s awareness about male and, specifically, female methods, including EC, varied mainly by their information and acquisition source awareness, but not recent FP outreach exposures. Among sexually active young men only, greater awareness across all methods was associated with pharmacy information and specific acquisition source awareness (i.e., public healthcare settings, mobile clinics, and faith-based organizations) and greater female method awareness only was associated with doctors/nurses as contraception information sources. Future work should work with these settings to review current approaches to engage all young men in contraception education and access, including young men not yet sexually active. Although recommendations for annual well-adolescent or routine screening visits for sexually transmitted infection testing are not in place for youth in SSA, some young men may learn about female methods when accompanying partners to the doctor/nurse or for their own sexual health-related problem visits. For all young men, greater female method awareness only was associated with health workers as information sources and awareness of FP clinics, nonprofit organizations, and fieldworkers as acquisition sources. These findings may be indicative of targeted FP outreach approaches of these setting types with young people, highlighting the important roles these settings can play as contraception information sources for young men.

Study findings highlight the value teachers play as young men’s preferred contraception information sources and its association with greater awareness across all methods for all young men. These findings may reflect the presence of CSE guidelines in all three countries [[28], [29], [30]]; however, CSE implementation has not been assessed. Furthermore, past reviews on school-based CSE interventions demonstrated varying inclusion of contraception information for male youth [31]; intervention studies that teach about FP have demonstrated increases in youths’ FP method knowledge, but did not stratify findings by sex [32]. Despite the current study’s findings that young men’s greater awareness of all methods was associated with friends, the Internet, and social media as information sources, young people do not always describe these sources as being trustworthy and may seek out other more reliable sources, such as healthcare providers [33]. Peer-led interventions have shown increased reproductive health knowledge among youth [34], but programs using peer educators may need to monitor the validity of peer-delivered intervention content and prevention messages [35]. Despite young men ranking mothers as preferred information sources, mothers (and fathers) as information sources were not associated with young men’s awareness about any method. Past work in SSA highlights the need for strategies to engage parents in open dialogues about sexual health with children due to lack of parental knowledge and skills and other cultural barriers and taboos about discussing sex [[36], [37], [38]].

This analysis has a number of limitations. First, cross-sectional findings are associative and not predictive. Next, the survey assessed self-reports of method awareness, information sources, outreach exposures, and acquisition sources that could be influenced by social desirability and recall bias. Future work should gain a better understanding of young men’s method knowledge and correct method use, content breadth and depth covered by their sources, including parents, barriers to method acquisition, use, or misinformation, and actual contraceptive acquisition behaviors that may also help to minimize some of the reporting biases. Future work should also explore contextualization of findings within each city and partners as an information source, since this was not the focus of the current study. Items assessing method awareness listed methods without providing accompanying explanations or definitions and outreach exposures did not define FP methods. It is possible that some respondents may be unaware of the method name, but still have basic awareness of the method. Method exposure also tapped into traditional FP methods, rather than more modern methods. The current analysis also did not assess fertility awareness-based methods. Future work should explore young men’s awareness of these methods, but reports on female adolescents’ use of these methods was low (ranging from 5% in Nairobi to 9% in Abidjan) [3,4,19]. Original data were collected using RDS from three cities in SSA and the sample, particularly in Abidjan, reported higher levels of education than the general population of youth in the cities, thus findings may not apply to all young men in these cities or from nonurban settings in the same countries. Future work should also explore how study findings vary by city context. A major strength of the current analyses is that it described for the first time young men’s male and female modern contraception method awareness, and contraception information sources, outreach exposures, and acquisition source awareness by sexual behavior status.

Findings demonstrate overall low rates of young men’s awareness about male and female methods and EC, except for condoms, information, and acquisition source awareness. Findings highlight specific information sources and settings that may be most relevant for program planners working with young men on contraceptive outreach and the importance of contraception availability through private and public sectors.
