# Supplementary material for: Males’ Awareness of Female and Male Contraception Methods, Information, Outreach, and Acquisition Locations in Abidjan, Côte d’Ivoire, Nairobi, Kenya, and Lagos, Nigeria
Source: J Adolesc Health. 2022 Sep;71(3):351–9. doi: 10.1016/j.jadohealth.2022.03.013 (PMC9365297; doi:10.1016/j.jadohealth.2022.03.013)
Supplement: Appendix Tables 1–5 [file mmc1.docx]

**Appendix**

**Appendix Table 1. Young men’s contraception method awareness by sexual behavior status for each city**

|  | **Abidjan, Côte d’Ivoire** | | | | | **Nairobi, Kenya** | | | | **Lagos, Nigeria** | | | | |
| --- | --- | --- | --- | --- | --- | --- | --- | --- | --- | --- | --- | --- | --- | --- |
| **Method awareness** | **Sexually**  **active** | | **Never**  **sexually active^a^** | | **Sexually**  **active** | | | **Never**  **sexually active^a^** | | **Sexually**  **active** | | **Never**  **sexually active^a^** | |  |
|  | **N^b^** | **%^c^** | **N^b^** | **%^c^** | **N^b^** | | **%^c^** | **N^b^** | **%^c^** | **N^b^** | **%^c^** | **N^b^** | **%^c^** |  |
| Male method |  |  |  |  |  | |  |  |  |  |  |  |  |  |
| Condom |  |  |  |  |  | |  |  |  |  |  |  |  |  |
| No | 64 | 12.2 | 32 | 13.1 | 19 | | 6.3 | 21 | 14.0 | 80 | 28.5 | 103 | 31.2 |  |
| Yes | 664 | 87.8 | 250 | 86.9 | 460 | | 93.7 | 191 | 86.0*** | 260 | 71.5 | 259 | 68.8 |  |
| Withdrawal |  |  |  |  |  | |  |  |  |  |  |  |  |  |
| No | 542 | 82.3 | 229 | 83.9 | 343 | | 74.7 | 186 | 87.2 | 292 | 90.0 | 323 | 89.8 |  |
| Yes | 186 | 17.7 | 53 | 16.1 | 136 | | 25.3 | 26 | 12.8*** | 48 | 10.0 | 39 | 10.2 |  |
| Female method |  |  |  |  |  | |  |  |  |  |  |  |  |  |
| LARC |  |  |  |  |  | |  |  |  |  |  |  |  |  |
| No | 484 | 69.6 | 140 | 56.0 | 240 | | 54.2 | 147 | 64.4 | 321 | 95.3 | 344 | 94.6 |  |
| Yes | 254 | 30.4 | 150 | 44.0*** | 239 | | 45.8 | 65 | 35.6*** | 22 | 4.7 | 19 | 5.4 |  |
| SARC |  |  |  |  |  | |  |  |  |  |  |  |  |  |
| No | 285 | 34.6 | 72 | 31.3 | 197 | | 43.6 | 116 | 62.2 | 293 | 86.6 | 310 | 85.3 |  |
| Yes | 453 | 65.4 | 218 | 68.7 | 282 | | 56.4 | 96 | 37.8*** | 50 | 13.4 | 53 | 14.7 |  |
| Emergency contraception |  |  |  |  |  | |  |  |  |  |  |  |  |  |
| No | 447 | 69.1 | 179 | 65.6 | 179 | | 39.3 | 147 | 74.1 | 310 | 92.4 | 343 | 95.1 |  |
| Yes | 281 | 30.9 | 103 | 34.4 | 300 | | 60.7 | 65 | 25.9*** | 30 | 7.6 | 19 | 4.9* |  |

^a^Chi-square comparison of specific method awareness by sexual behavior status within each city

^b^Unweighted; ^c^Weighted

**p*<0.05, ****p*<0.001

**Appendix Table 2. Contraception information sources associated with young men’s awareness of contraception methods by sexual behavior status**

| **Sexual behavior** | **Condom awareness** | | | **Withdrawal awareness** | | | **LARC awareness** | | | **SARC awareness** | | | | **EC awareness** | | |
| --- | --- | --- | --- | --- | --- | --- | --- | --- | --- | --- | --- | --- | --- | --- | --- | --- |
| **status & source** | **%^a^** | **aRR** | **(95% CI)^b^** | **%^a^** | **aRR** | **(95% CI)^b^** | **%^a^** | **aRR** | **(95% CI)^b^** | **%^a^** | **aRR** | **(95% CI)^b^** | | **%^a^** | **aRR** | **(95% CI)^b^** |
|  |  |  |  |  |  |  |  |  |  |  |  |  | |  |  |  |
| **Sexually active** |  |  |  |  |  |  |  |  |  |  |  |  | |  |  |  |
| Mother, No (Ref) | 88.2 | -- |  | 20.4 | -- |  | 33.9 | -- |  | 50.9 | -- |  | | 46.0 | -- |  |
| Yes | 87.2 | 1.35 | (0.79-2.32) | 21.0 | 1.30 | (0.80-2.10) | 34.3 | 1.67 | (1.06-2.63) | 50.5 | 1.29 | (0.82-2.02) | | 32.5** | 0.91 | (0.57-1.47) |
| Father, No (Ref) | 88.2 | -- |  | 21.0 | -- |  | 32.8 | -- |  | 49.6 | -- |  | | 44.3 | -- |  |
| Yes | 87.1 | 1.08 | (0.58-2.04) | 17.8 | 0.94 | (0.59-1.50) | 40.5 | 1.99 | (1.18-3.35)* | 57.7 | 1.47 | (0.86-2.52) | | 35.3 | 0.96 | (0.57-1.61) |
| Other relative(s), No (Ref) | 87.0 | -- |  | 18.8 | -- |  | 31.5 | -- |  | 48.3 | -- |  | | 41.5 | -- |  |
| Yes | 95.5 | 3.05 | (0.93-9.98) | 34.1** | 2.37 | (1.37-4.11)** | 54.0** | 2.87 | (1.56-5.31)** | 70.8** | 2.48 | (1.22-5.04)* | | 53.7 | 1.95 | (1.07-3.55)* |
| Brother(s), No (Ref) | 87.2 | -- |  | 19.2 | -- |  | 33.3 | -- |  | 47.9 | -- |  | | 42.0 | -- |  |
| Yes | 91.8 | 1.77 | (0.91-3.46) | 26.6 | 1.62 | (0.96-2.72) | 37.1 | 1.32 | (0.81-2.14) | 64.5** | 1.85 | (1.12-3.06)* | | 47.1 | 1.63 | (0.95-2.80) |
| Sister(s), No (Ref) | 87.5 | -- |  | 20.5 | -- |  | 34.0 | -- |  | 50.3 | -- |  | | 43.9 | -- |  |
| Yes | 91.3 | 1.69 | (0.80-3.57) | 20.6 | 1.09 | (0.58-2.05) | 34.0 | 1.14 | (0.62-2.08) | 54.3 | 1.01 | (0.59-1.73) | | 36.0 | 0.88 | (0.49-1.61) |
| Friend(s), No (Ref) | 82.1 | -- |  | 13.0 | -- |  | 24.0 | -- |  | 42.5 | -- |  | | 34.4 | -- |  |
| Yes | 93.9** | 2.88 | (1.47-5.65)** | 28.0*** | 2.33 | (1.49-3.64)*** | 43.9*** | 2.25 | (1.48-3.40)*** | 59.2*** | 1.96 | (1.33-2.88)** | | 51.4*** | 1.70 | (1.14-2.53)* |
| Doctor/nurse, No (Ref) | 85.9 | -- |  | 20.2 | -- |  | 30.2 | -- |  | 45.9 | -- |  | | 39.7 | -- |  |
| Yes | 95.9** | 3.14 | (1.34-7.35)** | 21.6 | 1.02 | (0.67-1.55) | 48.2*** | 1.94 | (1.28-2.93)** | 68.9*** | 2.09 | (1.37-3.18)** | | 54.6** | 1.76 | (1.13-2.76)* |
| Pharmacist, No (Ref) | 87.0 | -- |  | 18.7 | -- |  | 32.4 | -- |  | 47.9 | -- |  | | 41.0 | -- |  |
| Yes | 97.4* | 4.30 | (1.04-17.83)* | 37.7** | 2.59 | (1.51-4.43)** | 48.8** | 1.69 | (0.95-3.00) | 78.8*** | 3.04 | (1.65-5.59)*** | | 61.5** | 2.28 | (1.17-4.45)* |
| Health worker, No (Ref) | 86.4 | -- |  | 20.4 | -- |  | 28.2 | -- |  | 44.4 | -- |  | | 40.2 | -- |  |
| Yes | 95.0** | 1.96 | (0.87-4.45) | 20.7 | 0.95 | (0.59-1.53) | 58.8*** | 3.07 | (1.90-4.97)*** | 78.3*** | 3.02 | (1.86-4.92)*** | | 54.6* | 1.45 | (0.88-2.39) |
| Teacher, No (Ref) | 84.9 | -- |  | 17.9 | -- |  | 26.4 | -- |  | 42.5 | -- |  | | 39.1 | -- |  |
| Yes | 93.9*** | 2.37 | (1.30-4.31)** | 25.6* | 1.62 | (1.07-2.44)* | 48.7*** | 2.78 | (1.89-4.11)*** | 66.9*** | 2.09 | (1.46-2.98)*** | | 50.3** | 1.60 | (1.09-2.34)* |
| Religious leader, No (Ref) | 87.5 | -- |  | 19.9 | -- |  | 32.3 | -- |  | 49.9 | -- |  | | 41.9 | -- |  |
| Yes | 97.1* | 3.02 | (0.67-13.62) | 30.1 | 1.42 | (0.76-2.63) | 63.4*** | 2.94 | (1.59-5.42)** | 67.5* | 1.72 | (0.85-3.51) | | 60.3* | 1.45 | (0.67-3.11) |
| Internet/web, No (Ref) | 85.4 | -- |  | 16.5 | -- |  | 27.4 | -- |  | 43.2 | -- |  | | 37.3 | -- |  |
| Yes | 97.7*** | 4.56 | (1.69-12.31)** | 35.0*** | 2.40 | (1.54-3.75)*** | 58.1*** | 2.76 | (1.74-4.37)*** | 79.2*** | 4.71 | (2.90-7.66)*** | | 63.4*** | 1.87 | (1.17-2.99)** |
| Social media, No (Ref) | 85.1 | -- |  | 17.3 | -- |  | 28.0 | -- |  | 43.9 | -- |  | | 37.3 | -- |  |
| Yes | 97.6*** | 4.82 | (1.77-13.13)** | 31.0** | 1.86 | (1.18-2.94)** | 53.8*** | 2.25 | (1.44-3.51)*** | 74.0*** | 3.14 | (1.96-5.01)*** | | 61.5*** | 1.88 | (1.17-3.03)** |
| **Not sexually active** |  |  |  |  |  |  |  |  |  |  |  |  | |  |  |  |
| Mother, No (Ref) | 80.1 | -- |  | 13.2 | -- |  | 29.3 | -- |  | 39.1 | -- |  | | 20.4 | -- |  |
| Yes | 84.8 | 1.55 | (0.86-2.78) | 12.1 | 0.87 | (0.39-1.96) | 26.7 | 1.12 | (0.58-2.16) | 40.5 | 1.29 | (0.74-2.25) | | 25.6 | 1.53 | (0.77-3.07) |
| Father, No (Ref) | 81.3 | -- |  | 12.5 | -- |  | 29.3 | -- |  | 37.8 | -- |  | 21.2 | | -- |  |
| Yes | 80.6 | 0.97 | (0.37-2.54) | 16.1 | 1.27 | (0.58-2.75) | 25.4 | 0.73 | (0.38-1.42) | 49.2 | 1.26 | (0.60-2.64) | 24.1 | | 1.16 | (0.56-2.38) |
| Other relative(s), No (Ref) | 80.2 | -- |  | 12.6 | -- |  | 28.3 | -- |  | 37.2 | -- |  | 21.3 | | -- |  |
| Yes | 89.1 | 1.75 | (0.70-4.41) | 15.9 | 1.16 | (0.44-3.04) | 31.9 | 0.76 | (0.35-1.65) | 58.0* | 1.44 | (0.67-3.12) | 24.8 | | 0.86 | (0.39-1.86) |
| Brother(s), No (Ref) | 81.1 | -- |  | 14.0 | -- |  | 26.2 | -- |  | 37.1 | -- |  | 20.2 | | -- |  |
| Yes | 81.5 | 0.90 | (0.37-2.21) | 8.3 | 0.48 | (0.21-1.12) | 40.7 | 1.40 | (0.67-2.96) | 50.1 | 1.14 | (0.51-2.56) | 28.3 | | 1.30 | (0.55-3.05) |
| Sister(s), No (Ref) | 80.8 | -- |  | 12.5 | -- |  | 27.9 | -- |  | 38.1 | -- |  | 19.4 | | -- |  |
| Yes | 85.2 | 1.36 | (0.54-3.45) | 17.6 | 1.40 | (0.44-4.44) | 36.5 | 1.74 | (0.68-4.47) | 51.4 | 1.72 | (0.75-3.91) | 42.6** | | 3.40 | (1.57-7.37)** |
| Friend(s), No (Ref) | 76.1 | -- |  | 8.9 | -- |  | 24.5 | -- |  | 30.0 | -- |  | 16.0 | | -- |  |
| Yes | 89.7** | 2.57 | (1.44-4.59)** | 19.8** | 2.47 | (1.23-4.93)* | 35.8* | 1.38 | (0.77-2.50) | 55.1*** | 2.68 | (1.53-4.69)** | 30.9** | | 2.23 | (1.21-4.11)* |
| Doctor/nurse, No (Ref) | 77.3 | -- |  | 11.5 | -- |  | 29.4 | -- |  | 38.3 | -- |  | 20.0 | | -- |  |
| Yes | 95.5*** | 4.89 | (2.05-11.64)*** | 18.5 | 1.52 | (0.63-3.66) | 25.6 | 0.63 | (0.33-1.22) | 43.1 | 1.00 | (0.52-1.91) | 28.2 | | 1.09 | (0.50-2.34) |
| Pharmacist, No (Ref) | 81.0 | -- |  | 12.2 | -- |  | 27.6 | -- |  | 38.3 | -- |  | 20.7 | | -- |  |
| Yes | 86.7 | 1.07 | (0.31-3.60) | 29.2* | 2.95 | (1.03-8.45)* | 54.4* | 3.00 | (1.33-6.75)** | 64.0* | 1.48 | (0.52-4.21) | 40.7* | | 2.27 | (0.92-5.64) |
| Health worker, No (Ref) | 79.6 | -- |  | 11.2 | -- |  | 24.7 | -- |  | 34.0 | -- |  | 19.1 | | -- |  |
| Yes | 93.6* | 2.98 | (1.00-8.88) | 27.1** | 2.90 | (1.20-7.00)* | 60.3*** | 2.61 | (1.37-4.98)** | 82.0*** | 4.30 | (1.98-9.37)*** | 40.8** | | 1.91 | (0.92-3.97) |
| Teacher, No (Ref) | 72.0 | -- |  | 8.7 | -- |  | 15.2 | -- |  | 24.5 | -- |  | 11.3 | | -- |  |
| Yes | 92.9*** | 4.36 | (2.02-9.41)*** | 18.4** | 2.33 | (1.08-5.02)* | 46.1*** | 3.48 | (1.99-6.09)*** | 58.6*** | 3.28 | (1.95-5.53)*** | 34.7*** | | 3.30 | (1.78-6.14)*** |
| Religious leader, No (Ref) | 80.3 | -- |  | 12.3 | -- |  | 28.6 | -- |  | 38.0 | -- |  | 21.2 | | -- |  |
| Yes | 96.4* | 5.31 | (1.08-26.08)* | 24.8 | 2.11 | (0.65-6.91) | 31.4 | 0.68 | (0.24-1.90) | 63.6* | 1.78 | (0.60-5.32) | 27.1 | | 1.01 | (0.38-2.71) |
| Internet/web, No (Ref) | 79.6 | -- |  | 11.5 | -- |  | 27.4 | -- |  | 36.6 | -- |  | 19.0 | | -- |  |
| Yes | 95.7** | 5.22 | (1.44-18.94)* | 26.5* | 2.82 | (1.23-6.45)* | 41.6 | 2.53 | (1.09-5.88)* | 65.4** | 5.02 | (2.13-11.84)*** | 44.7** | | 3.48 | (1.69-7.13)** |
| Social media, No (Ref) | 78.8 | -- |  | 11.3 | -- |  | 26.2 | -- |  | 35.8 | -- |  | 19.2 | | -- |  |
| Yes | 98.7*** | 20.90 | (6.72-65.00)*** | 24.7* | 2.87 | (1.21-6.82)* | 47.5** | 2.12 | (0.90-4.98) | 66.1** | 3.59 | (1.59-8.09)** | 38.6** | | 2.67 | (1.27-5.64)* |

aRR=Adjusted Relative Risk; CI=Confidence Interval; RR=Relative Risk; FP=family planning

^a^Weighted log binomial regression models examined bivariate associations of each contraception information source with awareness about each method

^b^Weighted log binomial regression models examined associations of each contraception information source with awareness about each method, after adjusting for participants’ background characteristics (i.e., age group, school attendance, and city)

**p*<.05; ***p*<.01; ****p*<.001

**Appendix Table 3. Recent FP outreach exposure associated with young men’s awareness of contraception methods by sexual behavior status**

| **Sexual behavior** | **Condom awareness** | | | **Withdrawal awareness** | | | **LARC awareness** | | | **SARC awareness** | | | **EC awareness** | | |
| --- | --- | --- | --- | --- | --- | --- | --- | --- | --- | --- | --- | --- | --- | --- | --- |
| **status & FP exposure** | **%^a^** | **aRR** | **(95% CI)^b^** | **%^a^** | **aRR** | **(95% CI)^b^** | **%^a^** | **aRR** | **(95% CI)^b^** | **%^a^** | **aRR** | **(95% CI)^b^** | **%^a^** | **aRR** | **(95% CI)^b^** |
|  |  |  |  |  |  |  |  |  |  |  |  |  |  |  |  |
| **Sexually active** |  |  |  |  |  |  |  |  |  |  |  |  |  |  |  |
| FP community exposure, last year, No (Ref) | 89.7 | -- |  | 24.9 | -- |  | 35.7 | -- |  | 51.1 | -- |  | 44.7 | -- |  |
| Yes | 85.5 | 0.67 | (0.38-1.17) | 14.4** | 0.50 | (0.32-0.76)** | 31.2 | 0.76 | (0.51-1.12) | 49.6 | 0.96 | (0.63-1.46) | 40.5 | 0.74 | (0.48-1.16) |
| FP authority exposure, last year |  |  |  |  |  |  |  |  |  |  |  |  |  |  |  |
| Religious leader, No (Ref) | 87.3 | -- |  | 20.4 | -- |  | 32.9 | -- |  | 49.3 | -- |  | 41.2 | -- |  |
| Yes | 89.5 | 0.93 | (0.45-1.92) | 22.2 | 1.00 | (0.62-1.60) | 40.5 | 1.14 | (0.72-1.80) | 51.7 | 0.95 | (0.60-1.52) | 53.6* | 1.33 | (0.83-2.15) |
| Civic/community leaders, No (Ref) | 86.0 | -- |  | 18.7 | -- |  | 30.9 | -- |  | 47.5 | -- |  | 42.7 | -- |  |
| Yes | 93.5* | 2.10 | (0.99-4.47) | 27.7* | 1.55 | (0.94-2.56) | 46.7** | 1.87 | (1.17-2.98)** | 57.6 | 1.63 | (1.00-2.65)* | 48.8 | 1.02 | (0.62-1.69) |
| State or municipal leaders, No (Ref) | 88.4 | -- |  | 21.1 | -- |  | 35.2 | -- |  | 50.3 | -- |  | 45.0 | -- |  |
| Yes | 75.5 | 0.67 | (0.27-1.69) | 15.0 | 0.98 | (0.48-2.03) | 22.9 | 1.11 | (0.46-2.69) | 40.1 | 0.75 | (0.35-1.59) | 24.1* | 1.14 | (0.50-2.61) |
| Governmental official, No (Ref) | 88.5 | -- |  | 20.6 | -- |  | 32.0 | -- |  | 48.6 | -- |  | 43.5 | -- |  |
| Yes | 86.5 | 0.79 | (0.42-1.51) | 21.3 | 1.00 | (0.61-1.63) | 39.9 | 1.42 | (0.92-2.18) | 52.3 | 1.24 | (0.79-1.95) | 45.3 | 1.00 | (0.62-1.60) |
| FP media exposure, past months |  |  |  |  |  |  |  |  |  |  |  |  |  |  |  |
| Radio, No (Ref) | 88.1 | -- |  | 17.4 | -- |  | 29.5 | -- |  | 54.7 | -- |  | 37.9 | -- |  |
| Yes | 87.8 | 0.73 | (0.37-1.44) | 22.4 | 1.24 | (0.79-1.96) | 36.4 | 1.04 | (0.68-1.60) | 48.9 | 0.82 | (0.55-1.24) | 45.7 | 0.87 | (0.55-1.37) |
| Television, No (Ref) | 78.7 | -- |  | 12.5 | -- |  | 21.6 | -- |  | 47.0 | -- |  | 35.3 | -- |  |
| Yes | 91.7*** | 2.32 | (1.27-4.24)** | 23.7** | 1.89 | (1.11-3.23)* | 39.0*** | 1.75 | (1.08-2.82)* | 52.2 | 1.02 | (0.64-1.64) | 45.9* | 0.96 | (0.57-1.60) |
| Newspaper, No (Ref) | 90.7 | -- |  | 21.3 | -- |  | 28.8 | -- |  | 50.8 | -- |  | 41.3 | -- |  |
| Yes | 86.0 | 0.57 | (0.32-1.00) | 20.0 | 0.92 | (0.60-1.40) | 38.7* | 1.56 | (1.05-2.31)* | 50.0 | 1.07 | (0.72-1.57) | 44.5 | 1.03 | (0.68-1.56) |
| Brochure, flyer, No (Ref) | 89.1 | -- |  | 19.2 | -- |  | 32.3 | -- |  | 51.5 | -- |  | 40.7 | -- |  |
| Yes | 85.2 | 0.58 | (0.30-1.13) | 25.2 | 1.40 | (0.90-2.19) | 42.0* | 1.49 | (1.00-2.22)* | 53.2 | 1.11 | (0.74-1.66) | 50.7* | 1.45 | (0.93-2.27) |
| Mobile voice/SMS, No (Ref) | 90.1 | -- |  | 21.5 | -- |  | 37.3 | -- |  | 53.7 | -- |  | 47.9 | -- |  |
| Yes | 80.8** | 0.72 | (0.40-1.32) | 19.4 | 1.12 | (0.65-1.93) | 24.7* | 0.87 | (0.52-1.46) | 42.5* | 0.97 | (0.59-1.60) | 27.1*** | 0.63 | (0.36-1.08) |
| Poster/billboard, No (Ref) | 84.9 | -- |  | 15.8 | -- |  | 24.8 | -- |  | 44.5 | -- |  | 31.2 | -- |  |
| Yes | 89.4 | 1.05 | (0.58-1.88) | 23.0* | 1.34 | (0.88-2.05) | 39.5*** | 1.51 | (1.01-2.25)* | 54.5** | 1.27 | (0.86-1.89) | 50.0*** | 1.58 | (1.03-2.42)* |
| Social media site, No (Ref) | 86.2 | -- |  | 15.6 | -- |  | 25.6 | -- |  | 42.6 | -- |  | 36.6 | -- |  |
| Yes | 88.7 | 0.94 | (0.54-1.63) | 22.3 | 1.43 | (0.81-2.50) | 36.8* | 1.35 | (0.83-2.22) | 53.3** | 1.71 | (1.09-2.69)* | 45.6 | 0.97 | (0.60-1.57) |
| **Not sexually active** |  |  |  |  |  |  |  |  |  |  |  |  |  |  |  |
| FP community exposure, last year, No (Ref) | 81.2 | -- |  | 12.1 | -- |  | 28.7 | -- |  | 37.4 | -- |  | 18.4 | -- |  |
| Yes | 80.7 | 1.07 | (0.63-1.84) | 15.0 | 1.33 | (0.65-2.70) | 28.5 | 1.05 | (0.60-1.86) | 42.5 | 1.44 | (0.76-2.71) | 27.8 | 2.16 | (1.12-4.16)* |
| FP authority exposure, last year |  |  |  |  |  |  |  |  |  |  |  |  |  |  |  |
| Religious leader, No (Ref) | 79.3 | -- |  | 10.6 | -- |  | 26.2 | -- |  | 37.7 | -- |  | 19.6 | -- |  |
| Yes | 86.1 | 1.35 | (0.60-3.07) | 19.3 | 1.89 | (0.89-4.01) | 35.9 | 1.35 | (0.65-2.80) | 41.0 | 0.93 | (0.48-1.80) | 26.7 | 1.19 | (0.56-2.56) |
| Civic/community leaders, No (Ref) | 80.1 | -- |  | 13.0 | -- |  | 30.2 | -- |  | 36.9 | -- |  | 19.5 | -- |  |
| Yes | 84.9 | 1.24 | (0.53-2.90) | 12.8 | 0.96 | (0.42-2.21) | 23.5 | 0.58 | (0.29-1.18) | 44.8 | 1.60 | (0.81-3.14) | 28.4 | 1.52 | (0.71-3.28) |
| State or municipal leaders, No (Ref) | 81.1 | -- |  | 12.4 | -- |  | 28.6 | -- |  | 37.9 | -- |  | 20.9 | -- |  |
| Yes | 81.4 | 1.26 | (0.49-3.22) | 20.3 | 1.88 | (0.71-5.01) | 31.4 | 1.40 | (0.50-3.97) | 48.0 | 1.38 | (0.59-3.22) | 29.1 | 1.98 | (0.72-5.43) |
| Governmental official, No (Ref) | 82.4 | -- |  | 12.1 | -- |  | 27.1 | -- |  | 36.5 | -- |  | 19.9 | -- |  |
| Yes | 77.8 | 0.86 | (0.47-1.59) | 15.1 | 1.34 | (0.68-2.67) | 33.1 | 1.58 | (0.84-2.97) | 43.6 | 1.40 | (0.77-2.52) | 25.5 | 1.78 | (0.94-3.37) |
| FP media exposure, past months |  |  |  |  |  |  |  |  |  |  |  |  |  |  |  |
| Radio, No (Ref) | 82.1 | -- |  | 12.1 | -- |  | 29.2 | -- |  | 42.6 | -- |  | 20.8 | -- |  |
| Yes | 80.7 | 0.79 | (0.44-1.42) | 13.6 | 1.24 | (0.60-2.54) | 28.5 | 1.10 | (0.62-1.97) | 37.4 | 1.17 | (0.69-2.00) | 22.3 | 1.20 | (0.66-2.20) |
| Television, No (Ref) | 79.3 | -- |  | 9.7 | -- |  | 24.1 | -- |  | 39.3 | -- |  | 20.7 | -- |  |
| Yes | 81.9 | 1.09 | (0.59-2.01) | 15.2 | 1.84 | (0.96-3.55) | 31.9 | 1.57 | (0.81-3.03) | 40.8 | 1.13 | (0.62-2.05) | 22.6 | 1.10 | (0.54-2.26) |
| Newspaper/magazine, No (Ref) | 78.6 | -- |  | 12.4 | -- |  | 28.5 | -- |  | 34.7 | -- |  | 20.7 | -- |  |
| Yes | 84.2 | 1.75 | (0.98-3.10) | 14.3 | 1.33 | (0.69-2.55) | 29.9 | 1.21 | (0.69-2.12) | 46.4* | 2.15 | (1.20-3.83)* | 22.7 | 1.60 | (0.84-3.04) |
| Brochure, flyer, No (Ref) | 81.5 | -- |  | 12.8 | -- |  | 29.0 | -- |  | 38.5 | -- |  | 21.7 | -- |  |
| Yes | 83.3 | 1.31 | (0.70-2.44) | 16.2 | 1.37 | (0.68-2.73) | 27.9 | 1.13 | (0.59-2.16) | 40.3 | 1.16 | (0.63-2.13) | 24.2 | 1.38 | (0.67-2.87) |
| Mobile voice/SMS, No (Ref) | 81.4 | -- |  | 14.3 | -- |  | 29.5 | -- |  | 41.2 | -- |  | 20.0 | -- |  |
| Yes | 81.1 | 1.15 | (0.67-1.98) | 10.2 | 0.67 | (0.31-1.47) | 26.6 | 1.08 | (0.53-2.16) | 32.4 | 0.83 | (0.44-1.56) | 26.5 | 1.92 | (0.88-4.16) |
| Poster/billboard, No (Ref) | 76.7 | -- |  | 9.4 | -- |  | 22.4 | -- |  | 30.2 | -- |  | 17.1 | -- |  |
| Yes | 86.0* | 1.74 | (0.94-3.23) | 17.9* | 2.22 | (1.13-4.37)* | 37.5** | 1.89 | (1.05-3.41) | 50.9*** | 2.53 | (1.46-4.38)** | 26.9 | 1.80 | (0.99-3.26) |
| Social media site, No (Ref) | 83.1 | -- |  | 14.8 | -- |  | 28.7 | -- |  | 38.2 | -- |  | 24.1 | -- |  |
| Yes | 80.6 | 0.94 | (0.48-1.85) | 12.1 | 0.88 | (0.43-1.80) | 29.3 | 1.31 | (0.72-2.39) | 40.4 | 1.57 | (0.88-2.79) | 19.3 | 0.91 | (0.49-1.72) |

aRR=Adjusted Relative Risk; CI=Confidence Interval; RR=Relative Risk; FP=family planning

^a^Weighted log binomial regression models examined bivariate associations of each recent FP outreach exposure with awareness about each method

^b^Weighted log binomial regression models examined associations of each recent FP outreach exposure with awareness about each method, after adjusting for participants’ background characteristics (i.e., age group, school attendance, and city)

**p*<.05; ***p*<.01; ****p*<.001

**Appendix Table 4. Contraception acquisition source awareness associated with young men’s awareness of contraception methods by sexual behavior status**

| **Sexual behavior status** | **Condom awareness** | | | **LARC awareness** | | | **SARC awareness** | | | **EC awareness** | | |
| --- | --- | --- | --- | --- | --- | --- | --- | --- | --- | --- | --- | --- |
| **& acquisition source** | **%^a^** | **aRR** | **(95% CI)^b^** | **%^a^** | **aRR** | **(95% CI)^b^** | **%^a^** | **aRR** | **(95% CI)^b^** | **%^a^** | **aRR** | **(95% CI)^b^** |
|  |  |  |  |  |  |  |  |  |  |  |  |  |
| **Sexually active** |  |  |  |  |  |  |  |  |  |  |  |  |
| Private healthcare setting, No (Ref) | 83.5 | -- |  | 24.9 | -- |  | 39.9 | -- |  | 34.0 | -- |  |
| Yes | 98.1*** | 7.45 | (3.32-16.72)*** | 54.9*** | 2.86 | (1.86-4.40)*** | 75.5*** | 4.10 | (2.66-6.32)*** | 63.0*** | 2.50 | (1.58-3.94)*** |
| Public healthcare setting, No (Ref) | 77.5 | -- |  | 17.2 | -- |  | 34.8 | -- |  | 23.9 | -- |  |
| Yes | 97.7*** | 9.81 | (3.17-30.41)*** | 49.4*** | 2.66 | (1.73-4.07)*** | 65.4*** | 2.62 | (1.71-4.02)*** | 60.2*** | 2.26 | (1.44-3.53)*** |
| Family planning clinic, No (Ref) | 86.4 | -- |  | 27.4 | -- |  | 44.5 | -- |  | 37.5 | -- |  |
| Yes | 92.8 | 1.27 | (0.52-3.14) | 53.8*** | 2.44 | (1.56-3.83)*** | 69.4*** | 2.27 | (1.46-3.53)*** | 58.7*** | 1.77 | (1.13-2.78)* |
| Pharmacy, No (Ref) | 77.9 | -- |  | 22.6 | -- |  | 37.2 | -- |  | 28.9 | -- |  |
| Yes | 97.7*** | 9.73 | (4.73-20.01)*** | 45.0*** | 2.23 | (1.50-3.31)*** | 63.8*** | 2.73 | (1.84-4.07)*** | 56.2*** | 2.50 | (1.65-3.78)*** |
| Market/store, No (Ref) | 85.4 | -- |  | 30.1 | -- |  | 46.3 | -- |  | 38.6 | -- |  |
| Yes | 98.6*** | 9.60 | (2.42-38.06)** | 49.9*** | 1.89 | (1.22-2.94)** | 69.3*** | 1.94 | (1.24-3.03)** | 60.0*** | 2.06 | (1.30-3.25)** |
| Non-profit organization, No (Ref) | 87.4 | -- |  | 31.9 | -- |  | 49.3 | -- |  | 41.1 | -- |  |
| Yes | 100.0 | 1.00 | - | 75.1*** | 4.31 | (2.12-8.75)*** | 79.6** | 2.84 | (1.25-6.46)* | 76.3*** | 2.86 | (1.16-7.04)* |
| Fieldworker, No (Ref) | 87.0 | -- |  | 30.9 | -- |  | 48.2 | -- |  | 39.1 | -- |  |
| Yes | 98.4** | 4.28 | (0.75-24.42) | 65.4*** | 2.52 | (1.36-4.64)** | 76.2*** | 2.50 | (1.26-4.98)** | 81.0*** | 3.49 | (1.77-6.88)*** |
| Mobile clinic, No (Ref) | 87.0 | -- |  | 30.9 | -- |  | 48.6 | -- |  | 40.8 | -- |  |
| Yes | 99.7*** | 36.31 | (7.92-166.40)*** | 70.1*** | 4.09 | (2.24-7.49)*** | 74.9*** | 2.63 | (1.33-5.20)** | 66.5*** | 1.96 | (1.05-3.67)* |
| Faith-based organization, No (Ref) | 87.8 | -- |  | 33.5 | -- |  | 50.2 | -- |  | 42.4 | -- |  |
| Yes | 100.0** | 18.81 | (2.37-149.31)** | 69.2** | 4.04 | (1.37-11.90)* | 93.8*** | 10.91 | (3.20-37.24)*** | 72.3* | 3.76 | (1.13-12.51)* |
| Friend/relative, No (Ref) | 86.6 | -- |  | 31.2 | -- |  | 46.5 | -- |  | 40.2 | -- |  |
| Yes | 98.1*** | 6.32 | (2.08-19.21)** | 54.7*** | 2.23 | (1.30-3.84)** | 82.7*** | 4.77 | (2.76-8.22)*** | 62.6*** | 2.22 | (1.22-4.02)** |
| **Not sexually active** |  |  |  |  |  |  |  |  |  |  |  |  |
| Private healthcare setting, No (Ref) | 77.7 | -- |  | 24.0 | -- |  | 32.2 | -- |  | 17.6 | -- |  |
| Yes | 95.2*** | 4.75 | (1.86-12.16)** | 49.7*** | 2.26 | (1.19-4.28)* | 71.2*** | 4.59 | (2.22-9.50)*** | 39.5** | 2.22 | (1.08-4.56)* |
| Public healthcare setting, No (Ref) | 74.7 | -- |  | 21.5 | -- |  | 31.4 | -- |  | 15.9 | -- |  |
| Yes | 93.3*** | 3.73 | (1.42-9.78)** | 43.0** | 1.55 | (0.83-2.89) | 55.0*** | 1.88 | (0.97-3.65) | 33.0** | 1.45 | (0.76-2.77) |
| Family planning clinic, No (Ref) | 77.4 | -- |  | 23.2 | -- |  | 33.4 | -- |  | 16.1 | -- |  |
| Yes | 93.1** | 2.98 | (1.28-6.93)* | 47.6** | 2.58 | (1.38-4.82)** | 59.7** | 2.60 | (1.26-5.38)* | 40.9*** | 2.80 | (1.36-5.76)** |
| Pharmacy, No (Ref) | 74.0 | -- |  | 22.6 | -- |  | 27.7 | -- |  | 16.1 | -- |  |
| Yes | 96.3*** | 8.07 | (3.02-21.52)*** | 42.2** | 1.84 | (1.07-3.16)* | 65.5*** | 4.96 | (2.75-8.95)*** | 33.9** | 2.13 | (1.18-3.86)* |
| Market/store, No (Ref) | 78.8 | -- |  | 25.7 | -- |  | 35.4 | -- |  | 18.6 | -- |  |
| Yes | 98.5*** | 15.26 | (3.10-75.21)** | 53.8** | 2.38 | (1.15-4.95)* | 71.8*** | 4.33 | (2.00-9.41)*** | 47.6*** | 3.63 | (1.60-8.22)** |
| Non-profit organization, No (Ref) | 80.5 | -- |  | 27.9 | -- |  | 38.7 | -- |  | 21.2 | -- |  |
| Yes | 100.0*** | 1.00 | - | 66.0** | 5.60 | (1.67-18.79)** | 66.0* | 3.39 | (1.12-10.25)* | 44.7* | 2.39 | (0.81-7.12) |
| Fieldworker, No (Ref) | 79.7 | -- |  | 25.9 | -- |  | 35.8 | -- |  | 19.9 | -- |  |
| Yes | 98.2* | 13.40 | (1.41-127.61)* | 68.2*** | 4.35 | (1.56-12.09)** | 89.3*** | 19.37 | (6.20-60.48)*** | 46.5* | 3.62 | (1.07-12.26)* |
| Mobile clinic, No (Ref) | 81.1 | -- |  | 27.4 | -- |  | 38.2 | -- |  | 21.2 | -- |  |
| Yes | 76.0 | 0.46 | (0.07-3.20) | 52.9 | 1.94 | (0.50-7.60) | 59.0 | 1.59 | (0.43-5.91) | 29.8 | 1.04 | (0.33-3.25) |
| Faith-based organization, No (Ref) | 80.5 | -- |  | 28.6 | -- |  | 39.2 | -- |  | 20.8 | -- |  |
| Yes | 100.0 | 1.00 | - | 29.4 | 1.25 | (0.18-8.55) | 39.2 | 2.31 | (0.38-13.99) | 60.8 | 4.56 | (0.57-36.30) |
| Friend/relative, No (Ref) | 79.8 | -- |  | 26.2 | -- |  | 36.1 | -- |  | 19.6 | -- |  |
| Yes | 97.0** | 7.33 | (1.57-34.19)* | 64.3** | 4.32 | (1.75-10.67)** | 84.6*** | 8.58 | (2.42-30.42)** | 51.2** | 4.10 | (1.29-13.01)* |

aRR=Adjusted Relative Risk; CI=Confidence Interval; RR=Relative Risk; FP=family planning

^a^Weighted log binomial regression models examined bivariate associations of each contraception acquisition source awareness with awareness about each method

^b^Weighted log binomial regression models examined associations of each contraception acquisition source awareness with awareness about each method, after adjusting for participants’ background characteristics (i.e., age group, school attendance, and city)

**p*<.05; ***p*<.01; ****p*<.001

**Appendix Table 5. Background characteristics associated with young men’s awareness of contraception methods by sexual behavior status**

| **Sexual behavior status** | **Condom awareness** | **Withdrawal awareness** | **LARC**  **awareness** | **SARC**  **awareness** | **EC**  **awareness** |
| --- | --- | --- | --- | --- | --- |
| **& background characteristics** | **%^a^** | **%^a^** | **%^a^** | **%^a^** | **%^a^** |
| **Sexually active** |  |  |  |  |  |
| Age group |  |  |  |  |  |
| 15-17 (Ref) | 77.3 | 15.3 | 19.4 | 44.7 | 20.9 |
| 18-20 | 89.0** | 22.4 | 37.2*** | 53.9 | 48.4*** |
| 21-24 | 90.8** | 20.9 | 36.6*** | 50.6 | 46.4*** |
| Highest grade in school completed |  |  |  |  |  |
| Primary school or less (Ref) | 83.6 | 27.2 | 27.2 | 37.2 | 39.8 |
| Secondary | 85.8 | 15.9 | 30.0 | 50.1 | 35.7 |
| More than secondary | 94.4* | 28.3 | 45.5 | 57.0 | 60.6 |
| City location |  |  |  |  |  |
| Abidjan, Côte d’Ivoire (Ref) | 87.8 | 17.7 | 30.4 | 65.4 | 30.9 |
| Nairobi, Kenya | 93.7 | 25.3* | 45.8** | 56.4* | 60.7*** |
| Lagos, Nigeria | 71.5*** | 10.0* | 4.7*** | 13.4*** | 7.6*** |
| **Not sexually active** |  |  |  |  |  |
| Age group |  |  |  |  |  |
| 15-17 (Ref) | 77.3 | 8.3 | 24.9 | 35.6 | 15.9 |
| 18-20 | 83.9 | 18.6* | 31.5 | 40.1 | 26.9* |
| 21-24 | 82.9 | 13.4 | 31.1 | 44.2 | 24.4 |
| Highest grade in school completed |  |  |  |  |  |
| Primary school or less (Ref) | 88.6 | 13.0 | 13.0 | 12.7 | 27.6 |
| Secondary | 78.6 | 12.9 | 31.7 | 41.7* | 19.4 |
| More than secondary | 85.6 | 13.5 | 26.0 | 49.5** | 30.4 |
| City location |  |  |  |  |  |
| Abidjan, Côte d’Ivoire (Ref) | 86.9 | 16.1 | 44.0 | 68.7 | 34.4 |
| Nairobi, Kenya | 86.0 | 12.8 | 35.6 | 37.8*** | 25.9 |
| Lagos, Nigeria | 68.8*** | 10.2 | 5.4*** | 14.7*** | 4.9*** |

^a^Weighted log binomial regression models examined bivariate associations of each background characteristic with awareness about each method

**p*<.05; ***p*<.01; ****p*<.001
