# Supplementary material for: Males’ Awareness of Female and Male Contraception Methods, Information, Outreach, and Acquisition Locations in Abidjan, Côte d’Ivoire, Nairobi, Kenya, and Lagos, Nigeria
Source: J Adolesc Health. 2022 Sep;71(3):351–9. doi: 10.1016/j.jadohealth.2022.03.013 (PMC9365297; doi:10.1016/j.jadohealth.2022.03.013)
Supplement: Appendix Table 6 [file mmc2.docx]

**Appendix Table 6. Summary of factors associated with young men’s awareness of male and female contraception methods and emergency contraception by sexual behavior status, overall and by city^1^**

|  | **Male methods** | | | | | | | | | | | | | | | | **Female methods** | | | | | | | | | | | | | | | | | **Emergency**  **contraception** | | | | | | | |
| --- | --- | --- | --- | --- | --- | --- | --- | --- | --- | --- | --- | --- | --- | --- | --- | --- | --- | --- | --- | --- | --- | --- | --- | --- | --- | --- | --- | --- | --- | --- | --- | --- | --- | --- | --- | --- | --- | --- | --- | --- | --- |
|  | **Condoms** | | | | | | | | **Withdrawal** | | | | | | | | **LARC** | | | | | | | | | **SARC** | | | | | | | |  |  |  |  |  |  |  |  |
|  | **SA** | | | | **NSA** | | | | **SA** | | | | **NSA** | | | | **SA** | | | | | **NSA** | | | | **SA** | | | | **NSA** | | | | **SA** | | | | **NSA** | | | |
|  | **T** | **A** | **N** | **L** | **T** | **A** | **N** | **L** | **T** | **A** | **N** | **L** | **T** | **A** | **N** | **L** | | **T** | **A** | **N** | **L** | **T** | **A** | **N** | **L** | **T** | **A** | **N** | **L** | **T** | **A** | **N** | **L** | **T** | **A** | **N** | **L** | **T** | **A** | **N** | **L** |
| **Contraception information source** |  |  |  |  |  |  |  |  |  |  |  |  |  |  |  |  | |  |  |  |  |  |  |  |  |  |  |  |  |  |  |  |  |  |  |  |  |  |  |  |  |
| Mother |  | + |  |  |  |  |  |  |  |  |  |  |  |  |  |  | |  |  |  |  |  |  |  |  |  |  |  |  |  |  |  |  |  |  |  |  |  |  |  |  |
| Father |  |  | + |  |  |  | + |  |  |  |  |  |  |  |  |  | | + | + |  |  |  |  |  |  |  | + |  |  |  |  |  |  |  |  |  |  |  |  |  |  |
| Other relative(s) |  | + | + |  |  |  | + |  | + | + |  | + |  |  |  |  | | + | + |  | + |  |  |  | -- | + |  |  | + |  |  |  |  | + |  |  |  |  |  |  |  |
| Brother(s) |  |  | + |  |  |  |  |  |  |  |  | + |  |  |  |  | |  | + |  |  |  |  |  |  | + | + |  |  |  |  |  |  |  |  |  | + |  |  |  | + |
| Sister(s) |  |  | + |  |  |  | + |  |  |  |  | + |  |  | + |  | |  | + |  |  |  |  |  |  |  |  |  |  |  |  |  |  |  |  |  |  | + | + | + | + |
| Friend(s) | + | + |  | + | + |  | + |  | + |  | + | + | + | + |  |  | | + |  | + |  |  |  |  |  | + |  | + |  | + |  | + |  | + | + |  |  | + |  |  |  |
| Doctor/nurse | + | + |  |  | + | + | + |  |  |  |  |  |  |  |  |  | | + |  | + |  |  |  | + |  | + | + | + |  |  | + |  |  | + | + |  |  |  | + |  |  |
| Pharmacist/shop | + |  | + | + |  |  | + |  | + | + | + |  | + |  |  | + | |  |  |  |  | + | + |  |  | + |  | + |  |  |  |  | + | + | + |  |  |  | + |  |  |
| Health worker |  |  |  |  |  |  | + | + |  |  |  | + | + |  | + |  | | + | + | + |  | + |  | + |  | + | + | + |  | + | + |  | + |  |  |  |  |  |  |  |  |
| Teacher | + |  |  |  | + | + |  | + | + |  |  |  | + |  |  | + | | + | + | + |  | + | + | + |  | + | + | + |  | + |  | + | + | + | + |  |  | + |  | + |  |
| Religious leader |  |  | + |  | + |  | + | + |  |  |  |  |  |  |  |  | | + |  | + |  |  |  |  |  |  |  |  |  |  |  |  |  |  |  |  |  |  |  |  |  |
| Internet/web | + |  | + | + | + | + |  | + | + | + | + | + | + |  |  |  | | + |  | + | + | + | + |  | + | + | + | + | + | + | + |  | + | + |  | + | + | + |  | + | + |
| Social media | + |  | + | + | + | + | + | + | + | + |  |  | + |  | + |  | | + | + | + | + |  |  |  |  | + | + | + | + | + |  | + | + | + |  |  | + | + | + |  | + |
| **Recent FP outreach exposure** |  |  |  |  |  |  |  |  |  |  |  |  |  |  |  |  | |  |  |  |  |  |  |  |  |  |  |  |  |  |  |  |  |  |  |  |  |  |  |  |  |
| FP community exposure, last year |  |  |  | -- |  |  |  |  | -- |  | -- | -- |  |  |  |  | |  | -- |  |  |  |  |  |  |  |  |  |  |  |  | + | -- |  |  |  |  | + |  | + |  |
| FP authority exposure, last year |  |  |  |  |  |  |  |  |  |  |  |  |  |  |  |  | |  |  |  |  |  |  |  |  |  |  |  |  |  |  |  |  |  |  |  |  |  |  |  |  |
| Religious leader |  |  |  |  |  |  |  |  |  |  |  |  |  |  |  | + | |  |  |  |  |  |  |  | + |  |  |  |  |  |  |  |  |  |  |  |  |  |  |  | + |
| Civic/community leaders |  |  | + |  |  |  |  |  |  |  |  |  |  |  |  |  | | + | + |  |  |  |  |  |  | + | + |  |  |  |  |  |  |  |  |  |  |  |  |  |  |
| State or municipal leaders |  |  |  |  |  |  |  |  |  |  |  |  |  |  |  |  | |  |  |  |  |  |  |  |  |  |  |  |  |  |  |  |  |  |  |  |  |  |  |  |  |
| Governmental official |  |  |  |  |  |  |  |  |  |  |  |  |  |  |  |  | |  |  |  |  |  |  |  |  |  |  |  |  |  |  |  |  |  |  |  |  |  |  |  |  |
| FP media exposure, past months |  |  |  |  |  |  |  |  |  |  |  |  |  |  |  |  | |  |  |  |  |  |  |  |  |  |  |  |  |  |  |  |  |  |  |  |  |  |  |  |  |
| Radio |  |  |  | -- |  |  |  |  |  |  |  |  |  |  |  |  | |  |  |  |  |  |  |  |  |  |  |  |  |  |  |  |  |  |  |  |  |  |  |  |  |
| Television | + |  | + | + |  |  |  |  | + |  |  |  |  |  | + |  | | + |  | + |  |  | + |  | -- |  |  |  |  |  |  |  |  |  | + |  |  |  |  |  | -- |
| Newspaper/mag |  |  |  |  |  |  |  |  |  |  |  |  |  |  |  |  | | + |  |  |  |  |  |  |  |  |  |  |  | + |  | + |  |  |  |  |  |  |  |  |  |
| Brochure, leaflet |  |  |  | -- |  |  |  |  |  |  |  |  |  |  |  |  | | + |  | + |  |  |  |  |  |  |  |  |  |  |  |  |  |  |  |  |  |  |  |  |  |
| Voice/SMS mobile |  |  |  |  |  |  | + |  |  |  |  |  |  |  |  |  | |  |  |  |  |  |  |  | -- |  |  |  |  |  |  |  | -- |  |  |  |  |  |  |  |  |
| Poster / billboard |  |  |  |  |  | + |  |  |  | + |  | -- | + |  |  |  | | + |  | + |  |  | + |  | + |  |  |  |  | + | + | + |  | + |  | + |  |  | + | + |  |
| Social media site |  |  |  |  |  |  |  |  |  | + |  |  |  |  |  |  | |  |  |  |  |  |  |  |  | + |  |  |  |  |  |  |  |  |  |  |  |  |  |  |  |
| **Contraception acquisition source awareness** |  |  |  |  |  |  |  |  |  |  |  |  |  |  |  |  | |  |  |  |  |  |  |  |  |  |  |  |  |  |  |  |  |  |  |  |  |  |  |  |  |
| Private healthcare | + | + | + |  | + | + |  |  | N/A |  |  |  | N/A |  |  |  | | + | + | + | + | + | + |  |  | + | + | + |  | + | + | + |  | + | + | + |  | + | + |  |  |
| Public healthcare | + | + | + |  | + |  | + |  | N/A |  |  |  | N/A |  |  |  | | + | + | + |  |  | + |  |  | + | + | + |  |  |  |  |  | + | + | + |  |  |  |  |  |
| Family planning clin |  |  |  |  | + | + | + |  | N/A |  |  |  | N/A |  |  |  | | + | + | + |  | + |  | + |  | + | + | + |  | + |  | + | + | + | + |  |  | + |  | + |  |
| Pharmacy | + | + | + | + | + |  | + | + | N/A |  |  |  | N/A |  |  |  | | + |  | + |  | + | + |  |  | + | + | + |  | + |  | + | + | + | + | + |  | + | + |  |  |
| Market/store | + | + | + | + | + |  | + | + | N/A |  |  |  | N/A |  |  |  | | + | + | + | + | + | + |  |  | + | + | + |  | + |  | + |  | + |  | + | + | + | + |  |  |
| NPO |  | + | + |  |  | + | + |  | N/A |  |  |  | N/A |  |  |  | | + | + | + |  | + | + |  |  | + |  | + |  | + | + |  |  | + | + |  |  |  | + |  |  |
| Fieldworker |  |  | + |  | + |  | + |  | N/A |  |  |  | N/A |  |  |  | | + | + | + | + | + |  | + |  | + | + | + | + | + |  | + |  | + | + | + | + | + |  | + |  |
| Mobile clinic | + | + | + | + |  | + |  |  | N/A |  |  |  | N/A |  |  |  | | + | + | + |  |  | + |  |  | + |  | + |  |  | + |  |  | + |  |  |  |  | + |  | + |
| FBO/church | + |  | + |  |  |  |  |  | N/A |  |  |  | N/A |  |  |  | | + | + |  |  |  |  |  |  | + |  | + |  |  |  |  |  | + | + |  |  |  |  |  |  |
| Friend/relative | + |  |  |  | + |  | + |  | N/A |  |  |  | N/A |  |  |  | | + |  | + |  | + | + | + |  | + | + | + | + | + |  | + | + | + | + |  |  | + |  |  |  |

^1^See Appendix for results of weighted log binomial regression models examining associations of each contraception information source, recent FP outreach exposure, contraception location acquisition awareness with each modern method awareness, respectively, unadjusted and adjusted for participants’ background characteristics

FP=Family planning; SARC=Short-acting reversible method; LARC=Long-acting reversible method; NSA=Never sexually active; SA=Sexually active; N/A=not applicable

FBO=Faith-based organization; NPO=Non-profit organization

“+”=Positive relationship in adjusted log binomial regression models

“--“=Negative relationship in adjusted log binomial regression models
